# VacA, the vacuolating cytotoxin of *Helicobacter pylori*, binds to multimerin 1 on human platelets

**DOI:** 10.1186/1477-9560-11-23

**Published:** 2013-11-12

**Authors:** Kaneo Satoh, Toshiya Hirayama, Katsuhiro Takano, Katsue Suzuki-Inoue, Tadashi Sato, Masato Ohta, Junko Nakagomi, Yukio Ozaki

**Affiliations:** 1 Department of Clinical and Laboratory Medicine, Faculty of Medicine, University of Yamanashi, 1110 Shimokato, 409-3898 Chuo, Yamanashi, Japan; 2 Department of Bacteriology, Institute of Tropical Medicine, Nagasaki University, 1-12-4852-8523 Sakamoto,Nagasaki, Japan; 3 Department of First Internal Medicine, Faculty of Medicine, University of Yamanashi, 1110 Shimokato, 409-3898 Chuo, Yamanashi, Japan

**Keywords:** Platelet, VacA, Multimerin1, CD62P, ITP

## Abstract

Platelets were activated under the infection with H. pylori in human and mice. We investigated the role of VacA, an exotoxin released by *H. pylori* in this context. Acid-activated VacA, but not heated VacA, induced platelet CD62P expression. However, VacA reacted with none of the alleged VacA receptors present on platelet membranes. We therefore analyzed VacA associated proteins obtained through VacA affinity chromatography, using MALDI-TOF-MS. Multimerin1 was detected in two consecutive experiments, as the binding protein for VacA. Plasmon resonance confirmed their binding, and dot blot analysis revealed that the peptide sequence AA 321-340 of multimerin 1 is the binding site for VacA. In conclusion, we propose a new interaction between multimerin1 and VacA *,* which may give another insight into *H. pylori*-induced platelet activations under *H. pylori* infection.

## Introduction

Immune thrombocytopenia purpura (ITP) is an autoimmune disorder caused by increased platelet clearance by anti-platelet autoantibodies [1]. The prevalence of *Helicobacter pylori* (*H. pylori*) infection on ITP patients was 65% from 25 papers (in the range of 21.6% - 90.6%) [2]. In 1998, Gasbarrini et al. reported that successful eradication of *H. pylori* is followed by recovery of platelet counts in ITP patients infected with this bacterium [3]. An accumulating body of evidence suggests that the eradication of *H. pylori* is indeed effective in increasing the platelet count in nearly half of *H. pylori*–infected patients with ITP [2].

While the mechanism by which *H. pylori* induces thrombocytopenia remains largely undetermined, there are several lines of evidence to suggest that its infection activates platelets. Platelet aggregate formation was observed in mice infected with *H. pylori*[4]. *H. pylori* infection induces CD62P surface expression of platelets both in mice and humans [4], and its eradication led to a decrease in CD62P surface expression in ITP patients with *H. pylori* infection [5]. Although there are reports to suggest that the bacteria activate platelets by directly interacting with platelet membrane proteins [6], it appears more feasible that certain products of *H. pylori* circulate in blood stream to interact with platelets, since *H. pylori* nests in gastric mucosa where there could be no extensive interaction between the bacteria and platelets. In agreement with this hypothesis, *H. pylori* water extracts induce the formation of circulating platelet aggregates in rat gastric mucosal microvessels [7].

*H. pylori* is equipped with a set of remarkable pathogenic factors, including the cytotoxin-associated antigen (CagA) and the vacuolating toxin A (VacA). The type IV secretion system (T4SS) serves to translocate CagA directly into host cells [8]. When delivered into gastric epithelial cells via T4SS, *H. pylori* CagA perturbs host cell signaling and thereby promotes disturbance of epithelium cells and gastric carcinogenesis. By nature, it is an endotoxin, which is not secreted into the milieu. The vacuolating cytotoxin, VacA, induces cellular vacuolation in epithelial cells [9]. VacA is produced as a 140 kDa precursor protein and actively secreted from *H. pylori* by a type V autotransporter mechanism [10]. The 87-95 kDa mature toxin is generated by proteolytic cleavage of a 140 kDa precursor at the bacterial outer membrane [11,12]. Thus, we hypothesized that if *H. pylori* secretes some substance which interacts with platelets, VacA could be one of the most likely candidates.

In this study, we investigated the role of VacA in inducing platelet activation, and attempted to identify the target protein with which VacA interacts.

## Materials and methods

This study complied with the Declaration of Helsinki and was approved by the ethics committee of the University of Yamanashi. Informed consent was obtained from all patients and healthy volunteers prior to the index procedure.

### Materials

Glutathione S-transferase (GST)-fusion protein of multinerin 1 (GST-MMRN1) was purchased from Abnova Corporation (Taipei, Taiwan). Phycoerythrin (PE)–conjugated anti-CD62P and mouse IgG1 control were from BD Biosciences (CA, USA). HRP-conjugated goat anti-rabbit IgG were from Santa Cruz Biotechnology (CA, USA). Synthetic-peptides (Table 1) were made by Operon Biotechnologies (Tokyo, Japan) on our order. The 8% to 16% gradient gel (sodium dodecyl sulfate–polyacrylamide gel electrophoresis [SDS-PAGE]) was from TEFCO (Tokyo, Japan). Colloidal gold staining kit was from Bio-Rad Laboratories (CA, USA).

**Table 1:** Synthetic peptides sequence

1	IHTNQAESHTAVGRGVAEQQ (291-310)
2	VAEQQQQQGCGDPEVMQKMT (306-325)
3	MQKMTDQVNYQAMKLTLLQK (321-340)
4	TLLQKKIDNISLTVNDVRNT (336-355)
5	DVRNTYSSLEGKVSEDKSRE (351-370)
6	DKSREFQSLLKGLKSKSINV (366-385)
7	KSINVLIRDI (381-390)

### Study population

Patients, >18 years old, presenting with dyspepsia and/or other symptoms suggestive of peptic ulcer diseases were recruited for this study between January 1999 and September 2000 in the department of first internal medicine, University of Yamanashi Hospital.

Active *H. pylori* infection was assessed by ^13^C urea breath test (UBT) in all recruited patients. Eighteen patients were UBT test negative, and 17 patients were UBT test positive. *H. pylori* - infected patients underwent bacterial eradication with the standard triple therapy: amoxicillin, clarithromycin and lansoprazol for one week. Eradication was assessed by UBT 4 to 6 weeks after treatment. Complete eradication was observed with five patients.

### Preparation of purified VacA

The toxin-producing strain *H. pylori* ATCC49503 was used as the source of VacA for purification according to our published procedure [13]. In brief, VacA was precipitated from culture supernatant with 50% saturated ammonium sulfate. Precipitated proteins were dialyzed and then applied to an anti-VacA-specific IgG antibody affinity column. VacA was eluted from the affinity column under acidic conditions. VacA was activated by the acidic elution. VacA was inactivated on 95°C at 10 minutes (Heat-inactivated VacA; H-VacA). Purified VacA and H-VacA were stored at -80°C.

### Platelet preparation

Venous blood collected from healthy drug-free volunteers or patients was collected into 10% sodium citrate (3.8% sodium citrate, wt/vol). Platelet-rich plasma (PRP) was obtained after centrifugation at 150 g for 12 minutes. Washed platelets were obtained by centrifugation as previously described [14], using prostacyclin to prevent platelet activation during the isolation procedure.

### Pull-down with VacA-coated beads

Purified VacA was covalently coupled to cyanogen bromide (CNBr)–activated Sepharose 4B beads (Amersham Biosciences, Piscataway, NJ) according to the manufacturer’s instructions. Glycine-coated beads were used as a negative control. Surface proteins of washed platelets were labeled with biotin using ECL Protein Biotinylation System (Amersham Biosciences). One milliliter biotin-labeled or -unlabeled washed platelets (1 × 10^9^/mL) was lysed by an equal volume of 2 × ice-cold lysis buffer [14] and precleared by 200 μL Sepharose 4B (50% slurry) for 1 hour. After the detergent-insoluble debris was cleared by centrifugation at 15,000 g for 10 minutes, the supernatant was incubated with 200 μL VacA-bound or glycine-bound Sepharose 4B for 2 hours at 4°C. The beads were washed 5 times in 1 × lysis buffer, and proteins were eluted from the beads with 40 μL of SDS–reducing sample buffer and boiled for 5 minutes.

Protein digestion and mass spectrometric analysis was entrusted to Protein Research Network, Inc. (Yokohama, Japan), and the database search was performed.

### Flow cytometric analysis

PRP (2 × 10^8^/mL) were incubated with 120 nM VacA or H-VacA for 30 minutes followed by staining with anti-CD62P-PE or mouse IgG1-PE Platelet Control for 20 minutes at room temperature in the dark. Platelets were mixed with 1% paraformaldehyde in phosphate-buffered saline (PBS) for 20 minutes, and samples were analyzed with a FACScan flow cytometer and CellQuest software (Becton Dickinson, CA, USA).

### VacA-MMRN1 binding assay(Dot-blot)

Synthesized peptides (Table 1) corresponding to the amino acid sequence 291-390 of MMRN1 were dissolved in saline and 2.5 μL (5 mM) of each peptide was spotted on PVDF membrane. The membrane was air-dried, rinsed with three times with TBS-T (Tris-buffered saline containing 0.1% Tween 20) and incubated with 1% bovine serum albumin for 1 h at room temperature. The membrane was incubated first with 10 ng/mL of VacA, secondly with 100 ng/mL of anti-VacA polyclonal antibody and then with HRP-conjugated anti-rabbit antibody, respectively, for 1 h at room temperature with gentle agitation. It was washed three times with TBS-T after each incubation step. Antibody binding was visualized with ECL Western Blotting Detection Reagents (GE Healthcare, UK).

### Surface plasmon resonance measurement

Specific interactions between VacA and GST-MMRN1 were analyzed using BIAcore X system (BIAcore, Uppsala, Sweden) at 25°C. GST-MMRN1 was covalently coupled to CM5 chip (BIAcore) using an Amine Coupling Kit (BIAcore) according to the manufacturer’s instructions. Regeneration of the protein-coated surfaces was achieved by running 10 μL of 10 mM NaOH through the flow cell at the rate of 10 μL/min 2 times. A control surface was reacted with the amine coupling reagent in the absence of ligand and then blocked with ethanolamine. Various concentrations of VacA in PBS with 1 mM Ca^++^ was perfused over the control surface or immobilized GST-MMRN1 surfaces at a flow rate of 20 μL/min, and the resonance changes were recorded. The sensorgram of the immobilized GST-MMRN1 surfaces was subtracted from that of the control surfaces and the dissociation constants (KD) was determined using the BIAevaluation software (BIAcore).

### Statistics

Statistical analysis was performed using the paired Student’s t test. P values of less than 0.05 were taken as the minimum to indicate statistical significance.

## Results

### CD62P expression in platelets

We carried out a small-size clinical study at our university to evaluate the CD62P expression in platelets from patients with dyspepsia and/or other symptoms suggestive of peptic ulcer diseases. The CD62P expression in platelets was 1.5 ±3.9% in patients free from *H. pylori* infection (n = 18). However, its expression was significantly increased to 9.5 ±8.1% with *H. pylori* infection (n = 17, p < 0.001), and with those who underwent eradication successfully (n = 5), the CD62P expression was significantly lowered (1.9 ± 0.8%, p < 0.02). These findings were in good agreement with the previous reports [4,5] and we were led to assume that platelets are activated by the presence of *H. pylori*. Although there are reports to suggest that platelet aggregation is induced by the bacterial body of *H. pylori*, there has been no report to show the presence of circulating *H. pylori* in blood stream, apart from the gastric mucosa. Thus, it is likely that some molecules produced by *H. pylori* move into circulation, and activate platelets. In this line of reasoning, we next cultured *H. pylori*, and evaluated the effects of the culture supernatant on CD62P expression in platelets. As we expected, the culture supernants of *H. pylori* induced the expression of CD62P in platelets from healthy individuals (data not shown).

### The effects of VacA on platelet functions

Of the toxins produced by *H. pylori*, CagA, an endotoxin, is injected into the cytoplasm of target cells by the type IV secretory apparatus. We thus speculated that VacA, an exotoxin released by *H. pylori,* is responsible for the platelet-activating effects of the culture supernatant, and sought to evaluate the effects of VacA on platelet functions.

**Figure 1:**
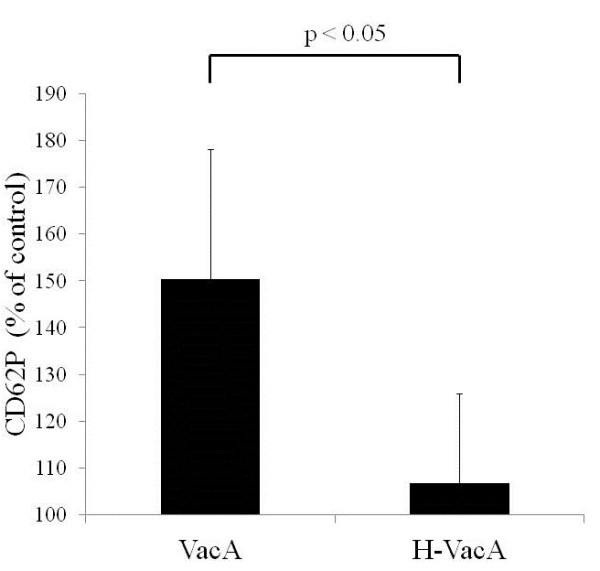
**VacA induces CD62P expression on platelets.** PRP were incubated with 120nM VacA or H-VacA. After 30 minutes, PRP were staining with anti-CD62PE or mouse control IgG1-PE for 20 minutes in the dark. Platelets were mixed with 1% paraformaldehyde in PBS, and samples were analyzed with a flow cytometer. The data expressed as per cent increase over the control are compiled from 6 experiments.

VacA did not induce platelet aggregation, when added to PRP (data not shown). However, it increased the expression of CD62P upon interaction with platelets (Figure 1). VacA, which is incubated at 95°C for 10 minutes, loses the vacuole-forming activity and the binding capacity to RPTPβ [15]. Heat-inactivated VacA (H-VacA) thus prepared did not significantly increase CD62P expression in platelets over the control, suggesting that VacA activates platelets by its biological activity, and that VacA interacts with its corresponding receptors on the platelet membrane for platelet activation.

### Exploration for the VacA receptor

The findings hitherto suggested that there is a specific receptor on the platelet membranes for VacA. Four cell surface proteins have been implicated so far as the specific receptors for VacA, including EGFR [16], RPTPα [17], RPTPβ [15,18] and CD18 [19]. CD18 is reportedly expressed in platelets [20]. However, the expression of RPTPα and RPTPβ, or EGFR, which is ordinarily expressed in the epithelial cell lineage, on the platelet membrane, remains unknown. We therefore evaluated the expression of these four VacA receptors on platelets by using the corresponding antibodies and Western blotting. We found that RPTPβ and CD18 were expressed on the platelet membrane. However, we were not able to detect the binding between VacA and each of these receptors with the use of either anti-VacA antibody or the antibody against each of these receptors. Based on these findings, we assumed that there is a receptor for VacA distinct from RPTPβ or CD18, which leads to platelet activation.

**Figure 2:**
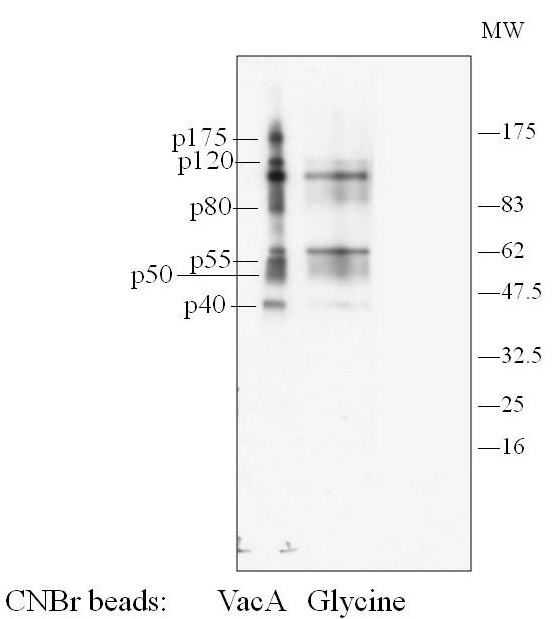
**Pull-down assay for detection of VacA binding protein(s).** Purified VacA was covalently coupled to CNBr-beads. Glycine-coated CNBr-beads were used as a negative control. Surface proteins in washed platelets were labeled with biotin. Platelets lysate was incubated with VacA bound or glycine bound beads. The beads were washed in lysis buffer, and proteins were eluted from the beads with SDS-reducing sample buffer. Eluted proteins were separated on SDS-PAGE, electroblotted, and proved with avidin-HRP (Figure 2) or with colloidal gold staining (data not shown). Left VacA beads, Right glycine beads.

**Figure 3:**
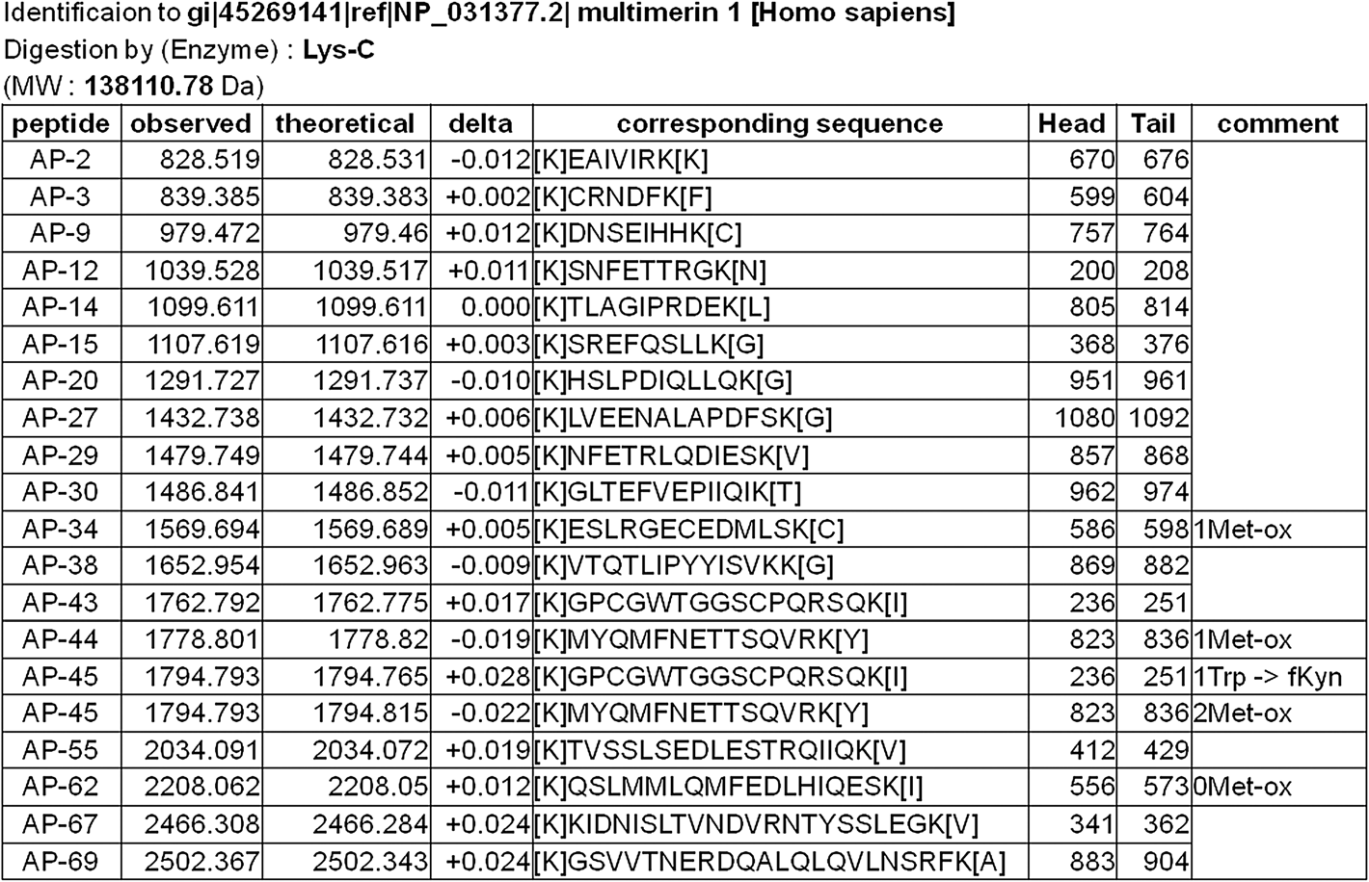
**Mass spectrometric analysis of p175.** p40, p50, p55, p80, p120 and p175 proteins in Figure 2 were excised and digested with endoproteinase Lys-C and analyzed by Mass spectrometry. The resulting peptide sequences matched with those of multimerin 1.

We next sought to identify the VacA-binding protein, using VacA affinity beads. Briefly, the proteins on the platelet membrane were biotin-labeled, and then solubilized by detergents. The lysates were reacted with VacA affinity beads, and after washing of the beads several times, the proteins were removed from the beads with the addition of SDS sample buffer. They were analyzed with SDS-PAGE, and after comparison with the glycine beads as the control, the protein bands specific for VacA affinity beads were identified. There were p40, p50, p55, p80, p120, and p175 bands, (Figure 2). All the bands were further analyzed by the peptide mass fingerprinting method, using MALDI-TOF-MS. As a result, a set of the peptides matched from the database suggested that multimerin 1 (MMRN1) was present in p175 (Figure 3). Previously reported VacA receptors (EGFR, RPTPα, RPTPβ, CD18) were not detected from any bands. The experiment was repeated twice with the same result, which suggested that the target protein for VacA on the platelet membrane is MMRN1.

**Figure 4:**
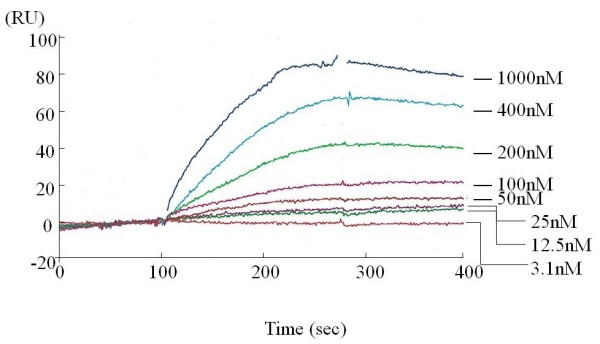
**Typical trace of GST-MMRN1-VacA association and dissociation.** GST-MMRN1 was covalently coupled to CM5 chip using an Amine Coupling Kit. A control surface was reacted with amine coupling reagent in the absence of ligand. Several concentrations of VacA was perfused over the control surface and immobilized GST-MMRN1 surfaces, and the resonance changes were recorded and analysed with a BIAcore system (n=2).

A GST-fusion protein of MMRN1 corresponding to the 291a.a. ~ 391a.a peptide sequence was commercially available. We checked with pulldown assay whether this GST-MMRN1 also reacted with VacA, which turned out to be the case. We utilized this protein for spotting the VacA-binding site with a Biacore based on the principle of surface plasmon resonance. First, we determined the binding affinity between VacA and GST-MMRN1, with GST-MMRN1 fixed on the measurement chip and various concentrations of VacA as eluate. There was a concentration-dependent binding (Figure 4), with the dissociation constant (KD) value of 3.3 x 10^-8^ (M), which suggests that VacA binds to MMRN1 with an affinity similar to that of antigen-antibody binding whose KD falls in the rage of 10^-7^ ~ 10^-9^ (M) [21].

**Figure 5:**
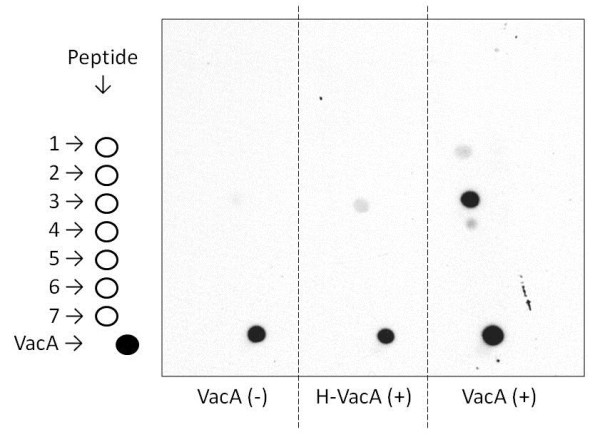
**VacA recognizes 321aa-340aa sequences.** Synthesized peptides (Table 1) were dissolved in saline, and spotted on PVDF membrane. VacA also spotted on PVDF membrane for the positive control. The membrane was incubated without VacA (VacA(-)), or with H-VacA or VacA. The membrane was incubated with anti-VacA polyclonal antibody and then HRP-conjugated secondary antibody (n=3).

Since the GST-fusion protein of MMRN1 corresponding to the 291a.a. ~ 391a.a peptide sequence is recognized by VacA, there should be the binding site for VacA within this sequence. We synthesized the 20-aa-length peptides, which partly overlap with one another (Table 1), and checked the binding of VacA with the dot blot method (Figure 5). As a result, the peptide sequence corresponding to 321a.a. ~ 340a.a. showed the highest reactivity to VacA, suggesting that VacA binds to MMRN1 by interacting with this region.

## Discussion

Recently, it has been suggested that *H. pylori* infection contributes to the pathogenesis of ITP, since partial or even complete remission of thrombocytopenia has been reported in a considerable portion of patients after eradication of *H. pylori*[2,22]. Some reports suggest the production of cross-reactive autoantibodies between the bacteria and platelets is responsible. In this line of reasoning, cross molecular mimicry between the *H. pylori* CagA protein or urease B, and platelet antigens has been implicated as a possible pathopysiological mechanism for this subset of ITP [23,24]. It was also suggested that autoreactive CD4^+^ T cells to glycoprotein IIb-IIIa (GPIIb-IIIa) mediate anti-platelet autoantibody production in patients with ITP. However, immediate recovery of platelet counts after bacteria eradiation may somewhat contradict the autoantibody production hypothesis, since it may persist for some time after the disappearance of the antigen(s). Another hypothesis which may better explain this phenomenon is that platelet activation and/or apoptosis induced by *H. pylori* itself and/or toxin(s) produced by *H. pylori*, leads to a decrease in platelet counts which then manifest as ITP. A report suggests that the bacteria activate platelets by directly interacting with GPIb, one of the major platelet membrane proteins [6]. However, it is more likely that certain products of *H. pylori* circulate in blood to interact with platelets, since *H. pylori* nests in gastric mucosa where there could be no extensive interaction between the bacteria and platelets. In this paper, we propose a new interaction between platelets and *H. pylori,* which may give another insight into *H. pylori*-induced ITP.

*H. pylori* infection induces CD62P surface expression of platelets both in mice and humans [4], a marker of platelet activation, and its eradication led to a decrease in CD62P surface expression in ITP patients with *H. pylori* infection [5]. However, the mechanism by which *H. pylori* induces platelet activation has remained unknown. In a series of preliminary experiments, we found that the culture supernatant of *H. pylori* induced the expression of CD62P in platelets, suggesting that some proteins (toxins) produced by *H. pylori* is responsible for this phenomenon. Since CagA, a most well-known toxin of *H. pylori* should be injected into the cytoplasm of target cells by a specific secretory apparatus, we speculated that VacA, an exotoxin released by *H. pylori*, plays a role of activating platelets, and sought to evaluate the effects of VacA on platelet functions.

VacA did not induce platelet aggregation, when added to PRP (data not shown). However, as we expected, it did induce the expression of CD62P upon addition to PRP. Heat-inactivated VacA, which is incapable of vacuole formation did not increase CD62P expression in platelets, suggesting that VacA activates platelets by its biological activity. These findings strongly suggest that there is a specific receptor on the platelet membranes for VacA. Four cell surface proteins have been implicated so far as the specific receptors for VacA, including EGFR, RPTPα, RPTPβ and CD18. We therefore evaluated the expression of these four VacA receptors on platelets by Western blotting, and detected RPTPβ and CD18 expression in platelets (data not shown). However, we were not able to detect the VacA-RPTPβ or VacA-CD18 binding with the use of either anti-VacA antibody, anti- RPTPβ or anti-CD18 antibodies. Thus, we drew a tentative conclusion that there is a receptor for VacA distinct from RPTPβ or CD18, which leads to platelet activation.

We sought to pinpoint the VacA-binding protein(s) on the platelet membrane with VacA affinity chromatography and the peptide mass finger printing method. As a result, two consecutive experiments both identified MMRN1 as a target protein for VacA. MMRN1 is a massive, homopolymeric adhensive protein, which belongs to the family of the Elastin-Microfibril Interface Located proteins (EMLIN). MMRN1 is the only EMILIN protein expressed in the megakaryocytic lineage, based on proteome and transcriptome analysis. It is stored in the secretion granules of megakaryocytes, platelets, and vascular endothelial cells [25]. Following platelet activation, MMRN1 is released from platelets and binds to activated platelet surface [26], external membranes of endothelial cells, and extracellular matrix.

Several lines of evidence have confirmed the interaction between VacA and MMRN1. A commercially available GST-fusion protein of partial MMRN1 (291aa-391aa) interacts with VacA, assessed by the far-western blot and the plasmon resonance analysis. We inquired of the supplier the reason why the GST fusion of protein of this specific sequence of MMRN1 was produced. However, we were not able to obtain relevant information. To the best of our knowledge, there has been no report to refer the structural or functional significance of the 291aa-391aa sequence of MMRN1. We speculate that the supplier estimated the most likely domain leading to the effective antibody production, using a computer software, and made the GST fusion protein encompassing 291aa ~ 391aa MMRN1 as the antigen. Commercial suppliers of reagents often follow this process in order to obtain antibodies to specific proteins. This hypothesis also implies that this site is externally exposed. Using several peptides corresponding to this sequence of MMRN1, we found that the 321aa ~ 340aa peptide of MMRN1 most strongly reacts with VacA, which suggests that VacA recognizes this sequence.

We hitherto found that VacA binds MMRN1, and that its binding site for VacA appears to reside within the 321aa ~ 340aa sequence. However, how this translates into VacA-induced platelet activation remains an issue. Although we have no direct evidence at present, several hypotheses could be postulated. MMRN1 is stored in platelet α granules, and is considered to be an activation marker, since it is released into extracellular milieu upon platelet activation. However, it is likely that at least a small amount of MMRN1 is already present on a certain population of platelets, which are partially activated or senescent, similar to platelet factor 4 (PF4). MMRN1 is known to bind αvβ3, αIIbβ3 and other unidentified receptors on the platelet membrane [27]. VacA, by binding to MMRN1, may facilitate the interaction between MMRN1 and these platelet activation receptors, with resultant activation of platelets. Platelet activation should lead to MMRN1 release and VacA-MMRN interaction, and with the formation of this positive feedback, more and more activated platelets may be sequestered with the onset of ITP.

Alternatively, the interaction between MMRN1 and the coagulation Factor V needs attention. MMRN1 is known to bind Factor V and activated Factor V with high affinity, and this binding appears to inhibit the coagulation cascade [28]. If VacA binding to MMRN1 should have some effects on Factor V inactivation by MMRN1, it may lead to enhanced thrombin generation, which would culminate in platelet activation.

Finally, similar to the pathogenesis of heparin-induced thrombocytopenia (HIT), in which the interaction between PF4 and heparin exposes a new epitope on PF4, thereby inducing the antibody production against the PF4-heparin complex, VacA and MMRN1 interaction may also induce the conformational change in MMRN1, exposing a new epitope. This process may lead to the production of antibodies, which activate platelets in a manner similar to that of HIT antibodies.

## Competing interests

None of the authors have any conflict of interest regarding this article.

## Authors’ contributions

KS, YO planed the concept, design, acquisition of the data. KT, KSI carried out the cell biological studies. TH, TS carried out the purification of materials. MO, JN contributed to the collection, and assembled the study data. YO critically revised the article. All authors read and approved the final manuscript.
